# The Structural Characterization of Tumor Fusion Genes and Proteins

**DOI:** 10.1155/2015/912742

**Published:** 2015-08-10

**Authors:** Dandan Wang, Daixi Li, Guangrong Qin, Wen Zhang, Jian Ouyang, Menghuan Zhang, Lu Xie

**Affiliations:** ^1^Institute of Food Science and Engineering, University of Shanghai for Science and Technology, Shanghai 200093, China; ^2^Shanghai Center for Bioinformation Technology, Shanghai Academy of Science and Technology, Shanghai 201203, China; ^3^Department of Cardiothoracic Surgery, The First Affiliated Hospital of People's Liberation Army General Hospital, Beijing 100048, China

## Abstract

Chromosomal translocation, which generates fusion proteins in blood tumor or solid tumor, is considered as one of the major causes leading to cancer. Recent studies suggested that the disordered fragments in a fusion protein might contribute to its carcinogenicity. Here, we investigated the sequence feature near the breakpoints in the fusion partner genes, the structure features of breakpoints in fusion proteins, and the posttranslational modification preference in the fusion proteins. Results show that the breakpoints in the fusion partner genes have both sequence preference and structural preference. At the sequence level, nucleotide combination AG is preferred before the breakpoint and GG is preferred at the breakpoint. At the structural level, the breakpoints in the fusion proteins prefer to be located in the disordered regions. Further analysis suggests the phosphorylation sites at serine, threonine, and the methylation sites at arginine are enriched in disordered regions of the fusion proteins. Using EML4-ALK as an example, we further explained how the fusion protein leads to the protein disorder and contributes to its carcinogenicity. The sequence and structural features of the fusion proteins may help the scientific community to predict novel breakpoints in fusion genes and better understand the structure and function of fusion proteins.

## 1. Introduction

**Figure 1:**
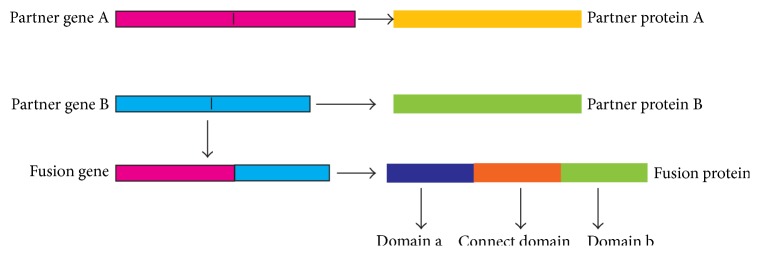
A multistep process of the formation of fusion proteins. A novel fusion gene is formed after two fusion partner genes A (pink) and B (blue) break at their breakpoints. The black points in the fusion partner genes and novel fusion gene represent the breakpoint. The fusion gene translates into a fusion protein, which has three domains, domain a (dark blue) from fusion partner protein a, domain b (cyan) from the fusion partner protein b, and a connection domain (orange).

Chromosomal translocations are commonly observed genomic abnormalities associated with hematological malignancies and sarcomas in human. Most chromosomal translocations in cancer involve reciprocal exchange of DNA between two chromosomes, resulting in the formation of novel fusion proteins [1]. The formation of a fusion protein includes multistep process (Figure 1). Two fusion partner genes (A and B) break at their breakpoints; after a series of reactions, two segments from the two separate genes join together, generating a novel gene, the fusion gene. Fusion genes then can be translated into fusion proteins (Figure 1). These fusion proteins contain functional domains that can activate or inhibit transcription, binding of DNA, or protein-protein interactions.

Chromosomal translocations are common in tumor [2]; however the mechanism of translocation is still poorly understood. Recent studies indicate translocations are not random. Environmental factors such as age and cigarette smoking might influence chromosomal translocation: the translocation frequency increases with age and the smokers have significant higher translocation frequency than nonsmokers [3]. Illegitimate V(D)J recombination, class switch recombination, homologous recombination, nonhomologous end joining, and genome fragile sites all have potential roles in production of nonrandom chromosomal translocations [4]. Chromosome spatial reposition in nuclear space is also responsible for nonrandom chromosomal translocations in human cancer [5]. Nonrandom chromosomal translocation may have internal influence factors of genes that form fusion genes. In this work, we studied the sequence feature of breakpoint in partner genes that form fusion genes in the genetic level.

Fusion genes function through translated fusion proteins. Previous study suggested that translocation-related human proteins are significantly enriched in disorder. The vicinity of the breakpoint is significantly more disordered than the rest of these already highly disordered fusion proteins [6]. The disordered regions are involved in important biological processes such as recognizing proteins, nucleic acids, and other types of partners. They accelerate interactions and chemical reactions between bound partners; and they help accommodate posttranslational modifications, alternative splicing, protein fusions, and insertions or deletions [7–9]. What contributes to the formation of disordered structure in fusion proteins and what is the effect of fusion protein structure on its function? These questions remain unresolved.

To address these questions, we explored the sequence features of partner genes and fusion genes, investigated the effect of breakpoint on irregular structure, and compared the posttranslational modification features on disordered domain and the structural domain of fusion proteins. Our results show that the breakpoints in the fusion partner genes have both sequence preference and structural preference. The breakpoints in the fusion proteins prefer to be located in the disordered regions. Further analysis suggests the phosphorylation sites and the methylation sites are enriched in disordered regions of the fusion proteins. Predicting the structure of EML4-ALK as an example, we explained how the fusion protein leads to the protein disorder and contributes to its carcinogenicity.

## 2. Material and Methods

### 2.1. Acquiring the Partner Genes, Fusion Proteins, and Breakpoints Associated with Cancers

The fusion gene information data in cancers is downloaded from the Cosmic database (http://cancer.sanger.ac.uk/cosmic/files?data=/files/grch38/cosmic/v73/CosmicFusionExport.tsv.gz) [10] and TicDB (http://www.unav.es/genetica/allseqs_TICdb.txt) [11]. The information of ID, breakpoint positions, and so forth of the partner genes is given in the Cosmic database. The sequences of partner genes are downloaded from the Ensembl database (http://www.ensembl.org/index.html) [12] filtered by the gene ID given in the fusion gene information data. Another source of fusion genes is from TICdb database. By blasting the nucleotide sequence from the TICdb database with the gene sequences in GenBank, the breakpoint information of partner genes was recorded. After deleting redundant genes, 192 partner genes which correspond to 427 breakpoints were collected.

To get the fusion protein sequences, we searched the human genomic and transcript database with the partial fusion genes from the TicDB database using Blastn method. The matching proteins (with query cover rate 100% and identity 100%) were selected. To more thoroughly collect fusion proteins, we further searched the mRNA sequence of fusion proteins in human Nucleotide database in NCBI using the key words of “fusion protein” and “chromosomal translocation.” The fusion genes with known breakpoints were selected. After removing redundant proteins, 128 fusion proteins with breakpoint were collected.

### 2.2. Calculating the Sequence Preference at the Breakpoint

Using the information of breakpoint of fusion partner genes and fusion genes, we extracted the nucleotide sequence with a length of ten residues around the breakpoints, namely, positions  *P* − 5, *P* − 4, *P* − 3, *P* − 2, *P* − 1, *P* + 1, *P* + 2, *P* + 3, *P* + 4, and *P* + 5. Here, − and + represent up- and downstream direction to the breakpoint. Nucleotide preference in each position around the breakpoint was calculated in the fusion partner genes and fusion genes, respectively. Specifically, the occurrence frequency of dinucleotide combinations of *P* − 1 and *P* + 1 is calculated in both fusion partner genes and fusion genes. Dinucleotide combinations from all human genes were used as the background comparison. The frequency of the trinucleotide combinations at *P* − 3, *P* − 2, and *P* − 1 was also calculated.

### 2.3. Analysis of the Structure of Breakpoints in the Fusion Proteins

The disorder tendency for each residue of the fusion proteins was predicted using IUPred algorithm [13]. As long sequences were reported to be more likely to form disorder structures, to achieve more accurate prediction, 108 fusion proteins with sequence length longer than 100 amino acids were selected for structural prediction [14]. A residue locating inside a disorder region was defined as beholding a disorder score larger than 0.5. To validate the prediction of IUPred, other prediction pieces of software, such as PreDisorder [15] and PONDR [16–18], were also used to predict the structure of the 108 fusion proteins.

### 2.4. Posttranslational Modification (PTM) Sites Prediction

We predicted the phosphorylation sites on fusion proteins using NetPhos 2.0 Server, which is a neural network-based tool for predicting potential phosphorylation sites [19]. All potential phosphorylation sites including tyrosine, serine, and threonine were predicted using the sequence of fusion proteins. Default threshold for the score of phosphorylation sites was used. The methylation sites were predicted using MeMo, which is based on support vector machine for predicting protein methylation [20]. The methylation sites focusing on arginine and lysine were predicted by input fusion protein names and its sequence in FASTA format. Using the information of predicted phosphorylation sites, methylation sites, and the structural prediction, phosphorylation and methylation preferences were calculated in disorder region, the structural domain, and the region around the breakpoint within the range of [−50,50] residues.

### 2.5. Structural Modeling of Fusion Protein EML4-ALK

Previously, we identified fusion proteins in ALK family from mass spectrometry data in lung cancer [21]. The EML4-ALK is a commonly observed fusion protein in non-small-cell lung cancer. The structures of nine isoforms of the EML4-ALK fusion proteins were predicted by IUPred. The function domains were annotated based on human protein database in NCBI. To understand the structural feature of EML4-ALK fusion proteins, we further predicted the structure of an EML4-ALK protein isoform, namely, EML4-ALK variant 1. EML4-ALK variant 1 contains 1059 amino acids, with breaking point at position 496 [22]. To build the structure of EML4-ALK variant 1 protein, we first performed blast of the EML4-ALK variant 1 sequence against the PDB database. Two crystal structures with the highest sequence alignment scores were found to be the crystal structure of the tandem atypical beta-propeller domain of EML1 (PDB id: 4CI8) [23] and the crystal structure of human anaplastic lymphoma kinase (PDB id: 4FOB) [24]. Swiss model online tool (http://swissmodel.expasy.org/) was used to build the structural model of EML4-ALK variant 1 protein using the template of 4CI8 and 4FOB [25]. All structures were presented by Pymol (http://www.pymol.org/).

## 3. Results and Discussion

### 3.1. Sequence Features of Breakpoints in Fusion Partner Genes

**Figure 2:**
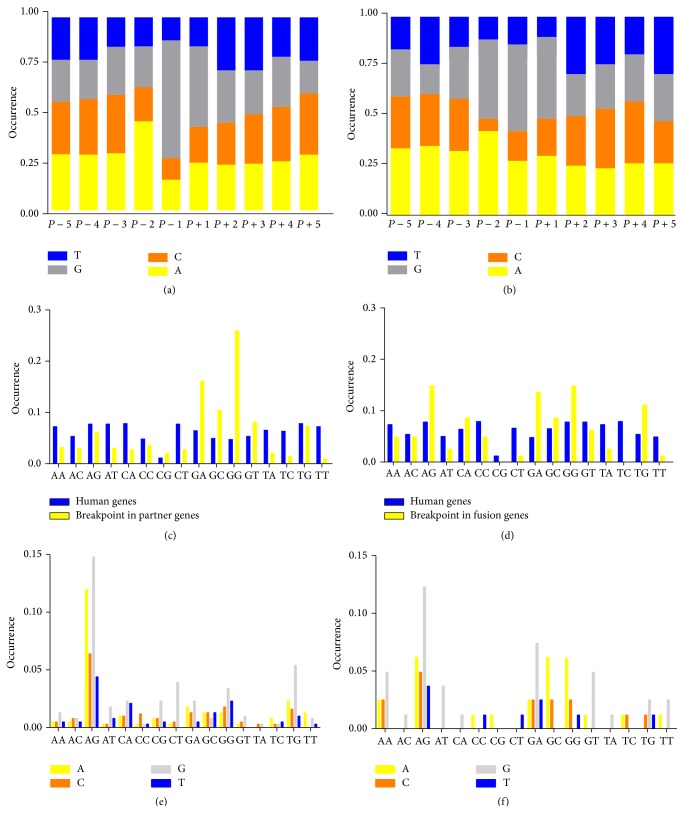
Nucleotide combinations of human genes and breakpoint in fusion genes. (a) Nucleotide preference at each site near the breakpoint in the partner genes. (b) Nucleotide preference at each site near the breakpoint in the fusion genes. ((c)-(d)) Frequency of dinucleotide occurrence at the breakpoint of partner genes (c) and fusion genes (d) compared to background human genes. (The blue line is the nucleotide combination of human genes, the occurrence of the combinations is similar, yellow line is the nucleotide combination of the breakpoint in fusion genes, and the occurrence of the combinations is different statistically.) ((e)-(f)) Sequence preference before the breakpoint in the partner genes (e) and fusion genes (f). The axis presents positions *P* − 2 and *P* − 1, and the different colors represent different nucleotide at *P* − 3.

There are a lot of factors that affect genetic disruption, such as the gene length and gene sequence features. Longer genes are easier to be fractured or inversed and gene sequences such as Alu sequences allow chromosomal rearrangements to be formed much easier. Strout et al. showed that partial tandem duplication which is one of the important mechanisms in acute myeloid leukemia was generated by Alu-mediated homologous recombination [26]. To investigate the sequence feature of the breakpoint in the partner genes, we calculated the sequence preference at the breakpoint using the 388 breakpoints from 163 fusion partner genes that associated with cancer. The nucleotide at the breakpoint prefers to be guanine according to our statistics (Figure 2). By calculating the single nucleotide preference near the breaking point of partner genes, we found that nucleotides G and A are preferred compared to T or C (Figure 2(a)). Particularly the nucleotide before the breaking point is dominantly occupied by G in the partner genes. In the fusion genes, nucleotide G is also preferred near the connection region (Figure 2(b)). Using the dinucleotide sequence distribution in all human genes as a background comparison, we found that, at the breaking point of partner genes, the occurring frequency of dinucleotides GG, GA, and GC is significantly higher than that in the background. Comparably, dinucleotide sequences TT, TC, and TA are much lower than total human gene background (Figure 2(c)). After the fusion of the genes, the frequency of GG in the connection points is lower than that in the partner genes (Figure 2(d)). The nucleotides before the breaking point also show nucleotide preference. At positions *P* − 1 and *P* − 2, nucleotide combination of AG is preferred (Figures 2(e) and 2(f)). Similar to the cleavage site of ALU sequence at AG/CT, the sequence at the breakpoint of fusion genes may be more easily recognized and cut by some enzymes that further contribute to gene interruption.

### 3.2. Structural Features of Fusion Proteins

**Figure 3:**
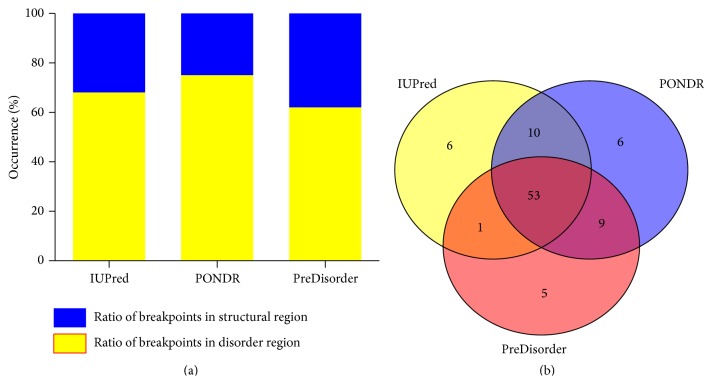
Prediction results of the breakpoints that fall into the disorder region predicted by IUPred, PONDR, and PreDisorder. (a) Ratio of breakpoints in structural region or disorder region predicted by the three pieces of software. (b) Venn diagram of the prediction amount of breaking points that located in the disorder region among the 108 breaking points.

The above studies indicate that the breakpoint positions in the fusion partner genes have sequence preference. As genes may function through being translated into proteins, to investigate the structure of fusion proteins, we further predicted the structures of the fusion proteins and the partner proteins. First, we collected 108 fusion proteins with known breakpoints in cancers and their partner proteins. Then, protein structure prediction pieces of software IUPred, PredDisorder, and PONDR were used to predict the protein irregularity. The ratios of breakpoints in disorder region are 68%, 72%, and 63% predicted by IUPRED, PONDR, and PreDisorder, respectively, which are quite similar (Figure 3(a)). Combining the results predicted by these three prediction pieces of software, 53 out of 108 fusion proteins were predicted to locate in the disorder region by all the three pieces of software (Figure 3(b)). The breakpoints of 70 fusion proteins that occupy 68% of the fusion proteins were predicted to be in the disorder region by at least two prediction pieces of software. Eighty-three percent of fusion protein breakpoints were associated with disorder region by at least one predictor. Comparably, before gene fusion, 52 percent of breakpoints in the products of partner genes fall into the disorder region, and 48 percent fall into the structural domain predicted by IUPred. These results show that the breakpoints in fusion proteins prefer to be in disorder region, and gene fusion may lead to the increasing of the disordered region compared to the partner proteins. To validate the prediction results, we also searched the structures of fusion proteins in the experimental structure database PDB. Although separate functional domains can be found, it is hard to find the connection region. As disordered protein sequences are much harder to be solved by X-ray crystallography, the results given in PDB database may support that the connection region prefers to be in the disorder region. Consistent with our results, previous study also indicated that the fusion protein contains a wealth of irregular regions and the structures near the breakpoint are significantly more disordered than the rest of these already highly disordered fusion proteins [6].

### 3.3. Posttranslational Modification in Fusion Proteins

**Figure 4:**
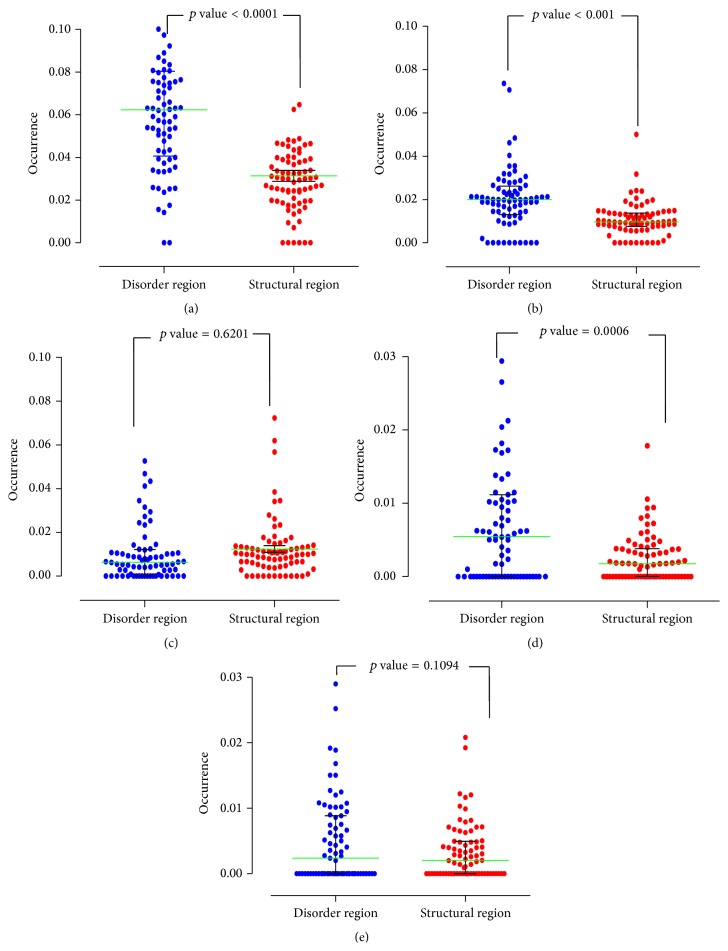
Predicted occurrence of posttranslational modifications in phosphorylation of serine, threonine, and tyrosine site and methylation of arginine and lysine in the intrinsic disorder area and the structural sequences. ((a)–(c)) Percentage of phosphorylation at the sites of the serine (a), threonine (b), and tyrosine (c) in the irregular area (blue) and in the structural sequences (red). ((d)-(e)) Percentage of methylation at the sites of arginine (d) and lysine (e) in the disorder region (blue) and in the structural sequences (red).

Although fusion proteins are well-known to have close relationship with tumor genesis, the mechanism of fusion proteins inducing tumor genesis is still poorly understood. Through the above study, we identified the sequence preference of the partner genes and fusion genes and the structure features of fusion proteins. How the structural feature influences the protein function needs to be addressed. Posttranslational modifications are essential for protein function. The abnormal protein posttranslational modification is usually associated with cancer. Importantly, posttranslational modification sites were reported to also prefer locating in disorder region [27, 28]. Here, we further calculated the posttranslational features of the fusion proteins. Protein phosphorylation and methylation are two frequently observed protein posttranslational modifications and they are important for the biological process. To investigate the relationship between the fusion protein structure and protein posttranslational modifications, we predicted the phosphorylation and methylation sites in either the structural region or the disordered region of fusion proteins. Protein phosphorylation may occur at positions of serine, threonine, or tyrosine residues, and methylation occurs at the residues of arginine and lysine. So we predicted the possible modification sites in each residue using the NetPhos 2.0 Server and MeMo online tool. The results reveal that phosphorylation modification sites at serine and threonine are more enriched in the disorder region than that in the structural region; however, tyrosine sites did not show significant difference (Figures 4(a)–4(c)). The arginine methylation sites in the disorder area are much higher than that in the structural region, but the lysine sites did not show the difference (Figures 4(d) and 4(e)). It is also supported by other studies that, in the irregular region, protein phosphorylation sites and methylation sites were preferred, but other posttranslational modifications, such as acetylation and glycosylation, are rare or nonexistent [27]. The results show that the phosphorylation modification and the methylation modification are enriched in the disorder area (Table 1). As the formation of fusion proteins may increase the disorder structure, the enrichment of posttranslational modifications in disorder region may promote the posttranslational modifications in the fusion protein.

### 3.4. The Mechanism of Carcinogenesis Induced by Fusion Protein in Lung Cancer

After characterizing of the sequence, structure, and posttranslational feature of the fusion protein, we further analyzed the mechanism of carcinogenesis induced by the fusion protein in lung cancer. The EML4-ALK is a commonly observed fusion protein in non-small-cell lung cancer. Nine isoforms of the EML4-ALK were collected in the present studies [29–31]. The partner genes are echinoderm microtubule-associated protein-like 4 (EML4) and anaplastic lymphoma kinase (ALK). EML4 contributes to the formation of the mitotic spindle and interphase microtubule network, and ALK is identified as a member of receptor tyrosine kinases which has oncogenic potential when its kinase activities are constitutively enhanced by rearrangement of the corresponding genes [32]. There are multiple breaking points in EML4 genes, but the ALK gene breaks at a more conserved site, which is near the N-terminal end of transmembrane domain (see Supplemental data, Figure  S1, in Supplementary Material available online at http://dx.doi.org/10.1155/2015/912742). In consistence with our statistical results above, the nucleotide combination AGG was frequently recognized in the breakpoint and a dinucleotide combination GG was the preferred cleavage site in EML4 gene. Comparing all the nine EML4-ALK fusion protein isoforms, the tyrosine kinase domains were retained; however different domains from EML4 were retained in different isoforms. The coiled-coil domain (CC) from EML4 which was predicted to be in the disorder region by all three predictors was retained for all EML4-ALK isoforms, which may mediate constitutive dimerization of EML4-ALK protein and contribute to tumorigenesis. The extracellular domain and the transmembrane domain of ALK protein were replaced by CC domain containing EML4 segment (Supplemental data, Figure  S1).

**Figure 5:**
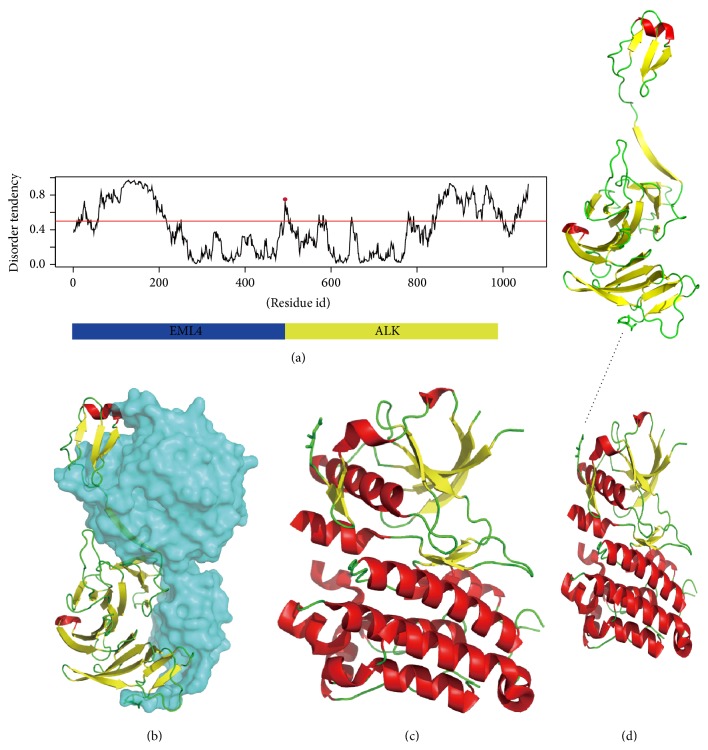
The structure characterization of EML4-ALK variant 1 fusion protein. (a) Disorder tendency of EML4-ALK variant 1 sequence predicted by IUPred. (b) The crystal structure of EML4 homology EML1, PDB id 4CI8. The subunits that form EML4-ALK variant 1 fusion protein are shown in cartoon, and the rest of the parts are shown in surface. (c) The crystal structure of the kinase domain of ALK protein that constitutes the C-terminal side of the EML4-ALK fusion protein. (d) The modeled EML4-ALK variant 1 protein by the structure of EML4 and ALK. A loop was added to connect the two parts.

In consistence with the above statistics, gene fusion of EML4-ALK protein increased the disorder tendency. Most of these breakpoints in the partner proteins were located in the structural region; however in the nine fusion protein isoforms, six breakpoints fell in the disorder region (Supplemental data, Figures  S1 and S2). We further analyzed the influence of disorder region with breakpoint on carcinogenesis by EML4-ALK variant 1 fusion protein as an example. The EML4-ALK variant 1 fusion protein contains the protein sequences from both EML4 protein and ALK protein. Two substructures were predicted to be connected by a disorder region (loop) (Figure 5(a)). As the normal function of ALK protein needs the dimerization triggered by signals from the extracellular domain, the fusion of EML4-ALK variant 1 containing a dimerization motif from EML4 might contribute to the dimerization of the EML4-ALK variant 1 and thereby trigger the autophosphorylation of the kinase domain and lead to the oncogenic potential in non-small-cell lung cancer [33]. The disorder region at the breakpoint of EML4-ALK variant 1 protein may facilitate the reunion of the two separate structure domains from two different partner proteins (Figures 5(b)–5(d)).

## 4. Conclusion

Gene fusion is a type of commonly observed genetic abnormity in the human genome, especially in cancer. However, the sequence and structure features of fusion genes and fusion proteins are still not fully understood. In this work, we explored the nucleotide preference at the breakpoint of fusion partner genes and fusion genes, predicted the secondary structure preference of fusion genes, and investigated the posttranslational modifications in the disordered fragment of fusion proteins. Several new sequential and structural features were discovered.

In the gene level, nucleotide combination of AG in front of the cleavage site is dominated in the partner genes and the dinucleotide combinations GG, GA, and GC are significantly higher than other combination compared to whole human genome. The sequence preference at the breakpoint of fusion genes may allow them to be more easily recognized and cut by some enzymes that further contribute to gene interruption. Our result may be helpful in predicting the location of the breakpoint in novel fusion partner genes.

In the structural level, our result suggests that the breakpoints in fusion proteins prefer to be in disorder region, and gene fusion may increase the disorder region in the fusion protein. Simultaneously, our statistics of posttranslational modification on fusion proteins show that the phosphorylation modification and the methylation modification are enriched in the disorder area. As the formation of fusion proteins may increase the disorder structure, the enrichment of posttranslational modifications in disorder region may promote the posttranslational modifications in the fusion protein, which further play roles in cancer.

Taking the EML4-ALK fusion protein as an example, we further summarized the sequence or structural features and the available experimental evidence to explain how this fusion gene might contribute to cancer carcinogenicity. The sequence features, structural preference, and posttranslational modifications may help others to predict the breakpoint site of fusion proteins and to predict the structure and function of fusion proteins, especially in cancer.

## Supplementary Material

The EML4-ALK is a commonly observed fusion protein in non-small cell lung cancer, we have collected nine isoforms of the EML4-ALK fusion proteins in the NCBI database. According to the “FEATURES” in the NCBI database we draw out the Supplemental data, Figure S1. The regions in the partner protein EML4 and ALK were compared to the nine fusion proteins to demonstrate the reserved domains in the fusion proteins. In order to look at the structure of the region with breakpoint and the reserved domains intuitively, the IUPred software was used to predicted the structure of nine isoforms, the result show in the Supplemental data, Figure S2.

## Figures and Tables

**Table 1:** The posttranslational modification sites in the disorder regions and the structural regions.

Posttranslational modifications sites	Ratio of occurrence at the disorder regions	Ratio of occurrence at the structural region
Serine	0.079	0.032
Threonine	0.024	0.011
Tyrosine	0.011	0.012
Arginine	0.009	0.003
Lysine	0.007	0.004
