# Supplementary material for: The Structural Characterization of Tumor Fusion Genes and Proteins
Source: Comput Math Methods Med. 2015 Aug 10;2015:912742. doi: 10.1155/2015/912742 (PMC4546970; doi:10.1155/2015/912742)
Supplement: Supplementary file 1 — The EML4-ALK is a commonly observed fusion protein in non-small cell lung cancer, we have collected nine isoforms of the EML4-ALK fusion proteins in the NCBI database. According to the “FEATURES” in the NCBI database we draw out the Supplemental data, Figure S1. The regions in the partner protein EML4 and ALK were compared to the nine fusion proteins to demonstrate the reserved domains in the fusion proteins. In order to look at the structure of the region with breakpoint and the reserved domains intuitively, the IUPred software was used to predicted the structure of nine isoforms, the result show in the Supplemental data, Figure S2. [file 912742.f1.docx]

**The Structural Characterization of Tumor Fusion Genes and Proteins**

Dandan wang^1,#^, Daixi li^1,*^, Guangrong qin^2,#^, Wen Zhang^3^, Jian Ouyang^1^, Menghuan zhang^2^, Lu Xie^2,*^.

^1^Institute of Food Science and Engineering, University of Shanghai for Science and Technology, Shanghai 200093, China.

^2^ Shanghai Center for Bioinformation Technology, Shanghai Academy of Science and Technology, Shanghai 201203, China.

^3^Department of Cardiothoracic Surgery, the First Affiliated Hospital of People Libration Army General Hospital, Beijing 100048, China

# These authors contribute equally to this work.

*To whom correspondence should be addressed:

Dr. Lu Xie, [xielu@scbit.org](mailto:xielu@scbit.org), Shanghai Center for Bioinformation Technology, Shanghai Academy of Science and Technology, Shanghai 201203, China.

Or Dr. Daixi Li, dxli75@usst.edu.cn, Institute of Food Science and Engineering, University of Shanghai for Science and Technology, Shanghai, 200093, China.

**Supplemental data**


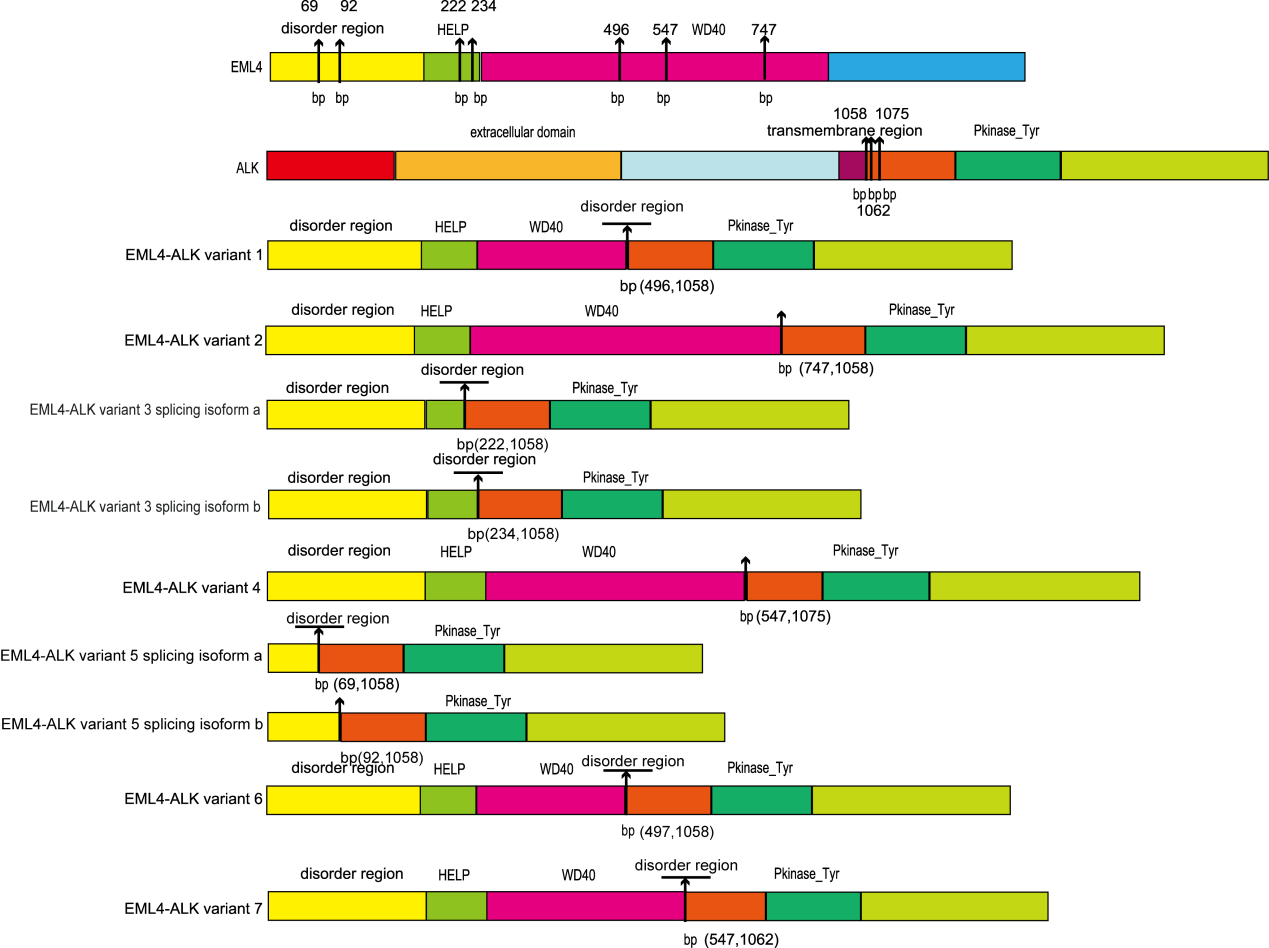


Supplemental data, Figure S1. Nine isoforms of the EML4-ALK fusion proteins. The abbreviated column identifier ”bp” is breakpoint.


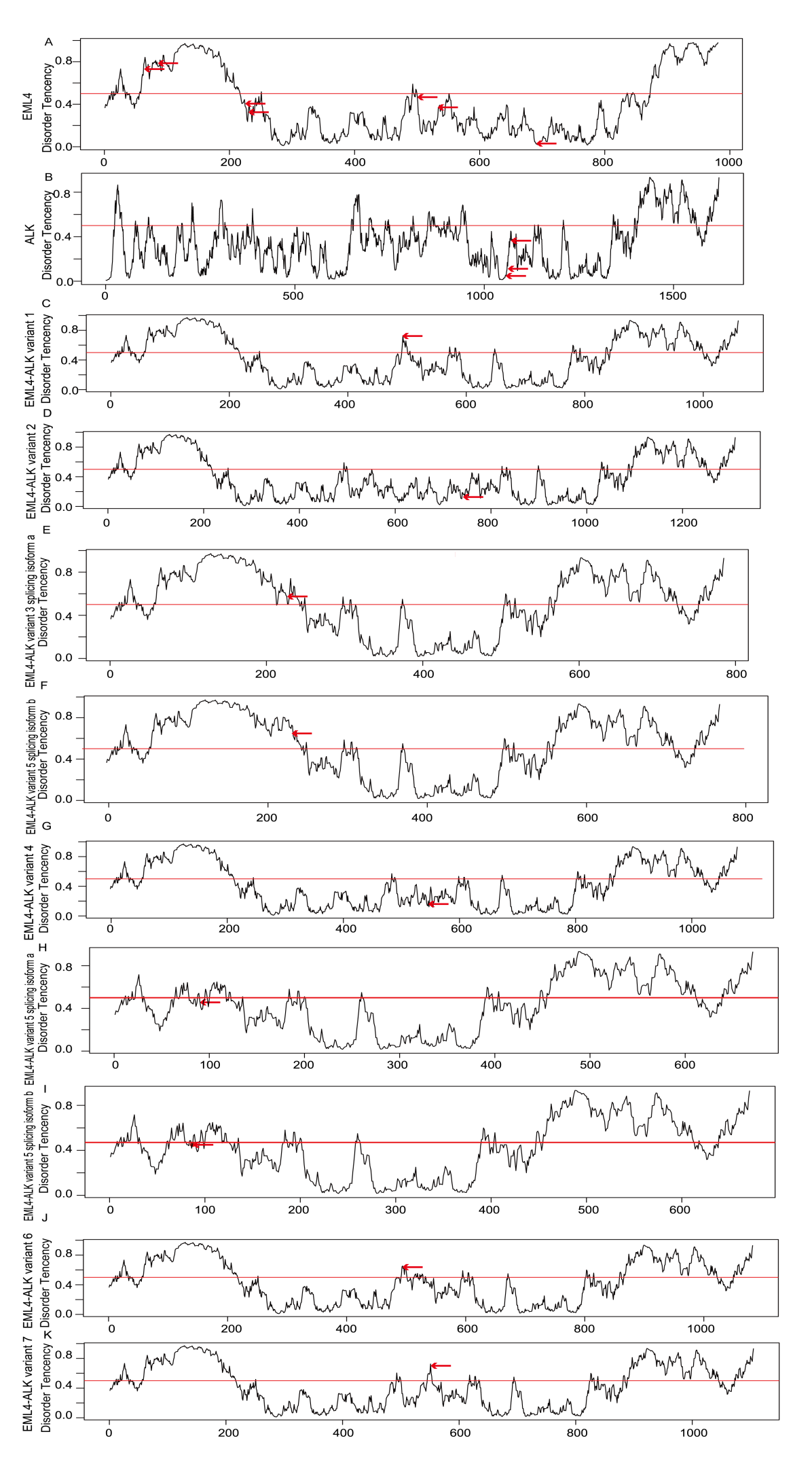


Supplemental data, Figure S2. The structure of nine isoforms of the EML4-ALK fusion proteins predicted by the software IUPred. The disorder regions are defined as the disorder tendency score > 0.5. The red arrow points to the breakpoint.
